# Morphological analysis of the vestibular system of guinea pigs poisoned by organophosphate^☆^^☆☆^

**DOI:** 10.1016/j.bjorl.2015.10.001

**Published:** 2015-10-24

**Authors:** Lícia Assunção Cogo, Valdete Alves Valentins dos Santos Filha, Adriana de Andrade Batista Murashima, Miguel Angelo Hyppolito, Aron Ferreira da Silveira

**Affiliations:** a Department of Physiotherapy, Universidade Federal do Pampa (UNIPAMPA), Rio Grande do Sul, RS, Brazil; b Department of Medicine, Sciences of Rehabilitation Program, Universidade de São Paulo (USP), São Paulo, SP, Brazil; c Universidade Federal de Santa Maria (UFSM), Santa Maria, RS, Brazil; d Department of Biology, Postgraduate Program in Ophthalmology, Otolaryngology, and Head and Neck-Shoulder, Universidade de São Paulo (USP), Ribeirão Preto, SP, Brazil; e Department of Medical Sciences, Universidade de São Paulo (USP), Ribeirão Preto, SP, Brazil; f Department of Ophthalmology, Otolaryngology, and Head and Neck-Shoulder, Universidade de São Paulo (USP), Ribeirão Preto, SP, Brazil; g Department of Veterinary Medicine, Universidade Federal de Santa Maria (UFSM), Santa Maria, RS, Brazil; h Department of Morphology, Universidade Federal de Santa Maria (UFSM), Santa Maria, RS, Brazil

**Keywords:** Postural balance, Vestibule labyrinth, Insecticides organophosphate, Pesticides, Toxicity, Equilíbrio postural, Vestíbulo do labirinto, Inseticidas organofosforados, Praguicidas, Toxicidade

## Abstract

**Introduction:**

The vestibular system is responsible for body balance. There are substances that damage it, causing dizziness; these are termed vestibulotoxic substances. Agrochemicals have been investigated for ototoxicity because of studies that identified dizziness as a recurrent symptom among rural workers’ complaints.

**Objective:**

To histopathologically evaluate the vestibular system in guinea pigs exposed to an organophosphate, and to identify the drug's effects on this system.

**Methods:**

Experimental clinical study. Eighteen guinea pigs were used; six of them poisoned with the organophosphate chlorpyrifos at doses of 0.5 mg/kg/day and seven of them at 1 mg/kg/day; and a control group of five guinea pigs was exposed to distilled water, all for 10 consecutive days. Later, ciliary tufts of saccule and utricle maculae were counted by scanning electron microscopy.

**Results:**

Comparing the groups, a one-way ANOVA test for the variable “saccule” (*p* = 0.0569) and a Kruskal–Wallis test for the variable “utricle” (*p* = 0.8958) were performed, revealing no difference among groups in both variables.

**Conclusion:**

The histopathologic analysis of the vestibular system of guinea pigs exposed to an organophosphate showed no difference in the amount of ciliary tufts of saccule and utricle maculae at the doses tested, although the result for the variable “saccule” was considered borderline, showing a trend for significance.

## Resumo

**Introdução:**

O sistema vestibular é responsável pelo equilíbrio corporal. Existem substâncias que o danificam, causando tontura; são chamadas vestibulotóxicas. Agrotóxicos tornaram-se objeto de investigação da ototoxicidade a partir de pesquisas que apontaram tontura como sintoma recorrente entre as queixas de trabalhadores rurais.

**Objetivo:**

Constitui-se em avaliar a histopatologia do sistema vestibular de cobaias expostas a organofosforados, identificando os efeitos nesse sistema.

**Método:**

É um estudo clínico experimental, que utilizou 18 cobaias, sendo seis intoxicadas com organofosforadoclorpirifós na dose de 0,5 mg/kg/dia; sete na dose de 1 mg/kg/dia, e grupo controle com cinco cobaias expostas a água destilada, durante 10 dias consecutivos. Posteriormente realizou-se a contagem dos tufos ciliares nas máculas dos sáculos e utrículos através microscopia eletrônica de varredura.

**Resultados:**

Na comparação intergrupos, para a variável sáculo realizou-se o teste ANOVA one-way (p = 0,0569); para a variável utrículo, utilizou-se o teste Kruskal-Wallis (p = 0,8958), revelando não haver diferença entre os grupos em ambas as variáveis.

**Conclusão:**

Análise histopatológica do sistema vestibular de cobaias expostas a organofosforado não demonstrou diferença na quantidade de tufos ciliares nas máculas dos sáculos e utrículos nas doses testadas, apesar do resultado para a variável sáculo ser considerado limítrofe mostrando uma tendência a significância.

## Introduction

The vestibular system consists of three main parts: a peripheral sensory system, a central processor, and a mechanism for motor responses. The peripheral sensory system consists of a set of motion sensors, which send information to the central nervous system (CNS). The CNS is responsible for processing these signals and for combining them with other sensory information, to estimate the cephalic orientation.^1^

This peripheral system consists of a bony labyrinth and a membranous labyrinth.^2^ The membranous component constitutes the functional part of the system, which detects balance sensations. This part contains three semicircular channels, a saccule and an utricle, constituting the vestibular sensory organs.^3^

The vestibular receptor cells are located in regions of the semicircular canals, called ampullae; when at the saccule and utricle location, these cells are called maculae. These cells are biological sensors, which convert the displacement caused by head movements into a neural discharge.^1^ However, these mechanical properties of the vestibular labyrinth provide its receptors the sensation of movement. The hair cells of the utricle and saccule register the linear movement. These organs have a gelatinous coating on the sensory cells of their maculae, with calcium carbonate crystals embedded in the surface of the gelatinous material lying on the sensory cells’ stereocilia. This gelatin undergoes a deformation caused by head movements, which divert the stereocilia.^3^

Body balance, for which the vestibular system is one of the responsible factors, is a complex interaction between sensory and motor systems that prevent the individual from falling, allow the adoption of different postures, and facilitate harmonious body movements.^4^ When a vestibular change occurs, symptoms of imbalance arise, including dizziness, which is the most common and can severely negatively impact the quality of life.^5^

There are substances that have the ability to damage or disrupt the vestibular system and cause dizziness. Such substances that negatively influence the system by causing loss of vestibular function or cell damage in the inner ear, are called ototoxic or vestibulotoxic substances.^6^ The degree to which these substances influence the patient depends on individual predisposition, administered dosage, duration of exposure, route of administration, age, family susceptibility, and/or possible prior damage to the inner ear.^7^

Several ototoxic substances have been evaluated, among them aminoglycoside antibiotics, aspartame, salicylic acid, oncological drugs, pesticides, and others.

Pesticides have become subject to investigation in the field of ototoxicity based on surveys of farmers, who pointed to dizziness and/or vertigo as recurring symptoms among complaints of this population.^8^, ^9^, ^10^, ^11^, ^12^ Studies reporting an ototoxic action and also neurototoxic effect of pesticides have been published. The neurototoxic effect derives from the inhibition of cholinesterase enzymes; this effect causes an interruption of propagation of nerve impulse to the body, giving rise to various symptoms and clinical signs.^13^

The ototoxicity of pesticides was reported in a survey involving 59 rural workers, of whom 49.15% had hearing changes.^14^ In a study conducted in a rural community on 33 employees, 54% of participants reported symptoms when pesticides were applied to plantations near the community where they live; among the symptoms, dizziness was reported by 12% of participants.^15^

These findings highlight the importance of studies of the vestibular system, in direct connection to the auditory system, and also taking into account findings in the literature that reveal clinical symptoms associated with the vestibular system, such as dizziness.

Human research allows the identification of symptoms, but does not allow for assessment of the morphological impact to the vestibular system of those who presents with these symptoms. Thus, the aim of this study was to conduct an analysis of the histopathology of the vestibular system of guinea pigs exposed to organophosphates, identifying the effects of these compounds on that system.

## Methods

This was an experimental study and was submitted to the Comissão de Éticaem Experimentação Animal (CETEA) of FMRP-USP (Faculdade de Medicina de Ribeirão Preto, Universidade de São Paulo), approved under protocol number 135/2011, and is in accordance with the ethical principles for animal experimentation adopted by the *Colégio Brasileiro de Experimentação Animal* (COBEA).

In this study, 18 adult male albino guinea pigs of the species *Cavia porcellus* weighing between 300 and 500 g were selected from the central vivarium of Biotério Central of Universidade de São Paulo, Ribeirão Preto campus. Guinea pigs positive for Preyer reflex, evaluated by observing small contraction movements of the ear pavilion in these animals, when the animal is stimulated with sounds of low and medium intensity, were included in this study. This reflex is used to evaluate auditory function in rodents.^16^ In the initial selection, the guinea pigs were assessed and submitted to manual otoscopy. Those animals showing signs of otitis externa and/or otitis media and of tympanic perforation were excluded from this experiment.

The animals were kept in the experimental surgery Unitat Biotério do Laboratório de Cirurgia Experimental, Department of Surgery and Anatomy of FMRP-USP (Faculdade de Medicina de Ribeirão Preto, Universidade de São Paulo) in separate collective cages containing wood shavings, according to experimental group, in a 12-h dark/light cycle, with an auto-feeding system and water *ad libitum*.

To conduct the study, guinea pigs were divided into three groups, with Group I as control;

– Group I: Five guinea pigs, with intraperitoneal administration of distilled water once daily; the same volume corresponding to that for the pesticide dose was used for 10 days, taking into account the weight of the animal.
– Group II: Six guinea pigs with intraperitoneal administration of pesticide in a single daily dose of 0.5 mg/kg/day for 10 consecutive days.
– Group III: Seven guinea pigs with intraperitoneal administration of pesticide in a single daily dose of 1 mg/kg/day for 10 consecutive days.

The 18 guinea pigs totaled 36 ears, with 36 saccules and 36 utricles.

The number of guinea pigs per group was established in accordance with the standards of the *Agência Nacional de Vigilância Sanitária*^17^ for conducting toxicology studies, with a minimum of five animals for each dose tested in the experiment.

The experimental model selected was the albino guinea pig, because according to a study by Albuquerque et al.^18^ this model proved to be the best animal in the microdissection stage, compared to rats. The advantages of this model are the size and strength of the temporal bone, which allows greater ease in removing anatomical material for analysis.

For guinea pig poisoning, the organophosphate Pyrinex 480 CE™ (Milenia Agrociências S/A) was administered. Pyrinex 480 EC™ is an organophosphate insecticide that acts by contact and ingestion, recommended for the control of pests in cotton, potato, coffee, citrus, bean, apple, corn, soybean, and creeping tomato for industrial purposes, and for wheat crops. The product is registered at the *Ministério da Agricultura*, *Pecuária e Abastecimento* (MAPA)^19^ under No. 09298.

The dose of the pesticide chosen was based on oral LD_50_ for rats with Pyrinex 480 CE™, found in the product package insert, which amounts to 300 mg/kg; the norms of ANVISA (not to exceed 80% of DL_50_) were followed. Guinea pigs had their weight monitored daily for proper calculation of the dose of the pesticide being used.

For guinea pig handling and administration of pesticides, personal protective equipment was used, as recommended in the product package insert.

After 24 h of the last administration of the pesticide, all animals were intraperitoneally anesthetized with thionembutal (25 mg/kg) at a dose 6 mg/kg body weight and were euthanized by decapitation. Immediately after the animals were sacrificed, the temporal bones with their tympanic bullae were removed. Tympanic bullae were opened and vestibular organs were removed.

Subsequent to the animals’ euthanasia, the anatomical specimens were perfused with a 3% glutaraldehyde fixation solution at 4 °C and maintained in this solution for 24 h. Through the round window, a 3% glutaraldehyde solution in 0.1 M phosphate buffer, pH 7.4, was injected for fixation, remaining there for 4 h at 4 °C; the specimens were washed three times for 5 min with the same buffer. Next, they were fixed with 1% osmium tetroxide for 2 h at 4 °C and then subjected to dehydration.

Dehydration of the structures was performed with successive ethanol baths in increasing concentrations of 50%, 70%, 90%, and 95% for 10 min each at room temperature. Then, 100% ethanol was used in three 20-min baths each, leaving the structures immersed in the last bath for 12 h at room temperature. The task of drying the water still present in the samples after dehydration was carried out using the equipment Bal-Tec CPD 030 – Critical Point Dryer (Balzers, Liechtenstein) using a critical-point process, and the samples were transferred to the apparatus chamber and covered with fluid carbon dioxide (CO_2_).

For proper observation under scanning electron microscopy (SEM), the dissected and partially prepared pieces were fixed on a metallic cylindrical sample holder with conductive carbon paste, Colloidal Graphite (Electron Microscopy Sciences – Hatfield, PA, United States). The structures were covered by a thin layer of 24-carat gold with the use of a vaporization process equipment, Bal-Tec SCD 050 – Sputter Coater (Balzers, Liechtenstein), becoming electrically conductive.

Morphological analysis of vestibular structures was performed by scanning electron microscopy in a JEOL™ JSM-5200 Scanning Electron Microscope. The images were photographed (3500× magnification). After the photographic register, the pictures were transferred to the ImageJ™ software, in order to carry out the evaluation.

A microscopic evaluation of ciliary tufts of saccule and utricle maculae was conducted bilaterally for each animal in the experiment. This assessment was made by counting the number of ciliary tufts in the striola area present in the photographic field. The 3500× magnification was standardized for all photographs.

For statistical analysis, the weighted average of the number of stereocilia tufts present in each ear was calculated. Those vestibular organs which, under SEM, showed artifacts that could interfere with ciliary tuft count, for instance, structural injury during dissection or assembly of the specimen, were excluded.

Data obtained were statistically analyzed by Statistica software, version 9.0, with a significance level set at 5%. Data normality was tested by the Lilliefors test. A descriptive analysis was performed. According to the result of normality test, normal data were compared between groups by an one-way ANOVA, and non-normal data were compared through Kruskal–Wallis test.

## Results

At the end of the analyses, six saccules and three utricles were lost, which were considered in “*n*” items in tables.

The Lilliefors test showed normality of data for the “saccule” variable and non-normality of data for the “utricle” variable. Descriptive analysis was performed for the number of saccule and utricle ciliary tufts in Groups I, II, and III, as shown in Table 1.

**Table 1:** Comparison of mean values of the number of saccule and utricle ciliary tufts among Groups I, II, and III.^a^

Group	Mean	Minimum	Maximum
*Group I*			
Saccule (*n* = 9)	25.44 ± 6.50	14.00	35.00
Utricle (*n* = 10)	24.86 ± 3.51	17.00	28.00

*Group II*			
Saccule (*n* = 9)	26.70 ± 5.54	20.00	38.00
Utricle (*n* = 11)	26.45 ± 4.50	18.00	34.00

*Group III*			
Saccule (*n* = 12)	31.41 ± 5.35	22.50	42.00
Utricle (*n* = 12)	27.97 ± 3.77	23.66	37.50

*n*, number of saccules and utricles.

a Data were presented as mean ± standard deviation.

To compare intergroup data for the “saccule” variable, a one-way ANOVA test was carried out, resulting in *p* = 0.0569, showing no difference between values of Groups I, II, and III for the variable tested. As for “utricle” variable, the comparison among groups was analyzed using the Kruskal–Wallis test, with *p* = 0.8958, showing no difference among groups.

**Figure 1:**
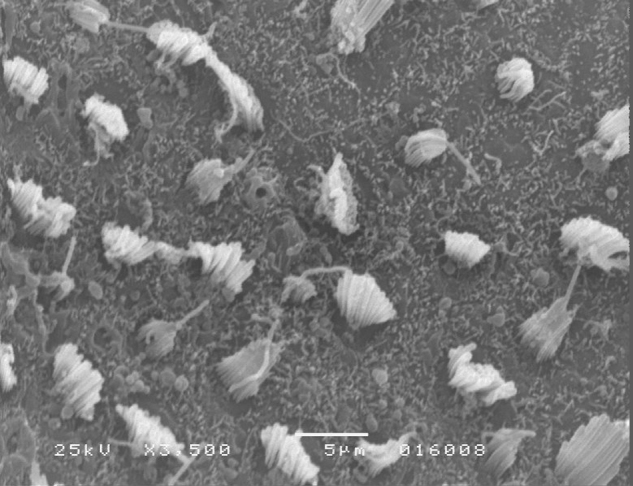
Utricle – Group I.

**Figure 2:**
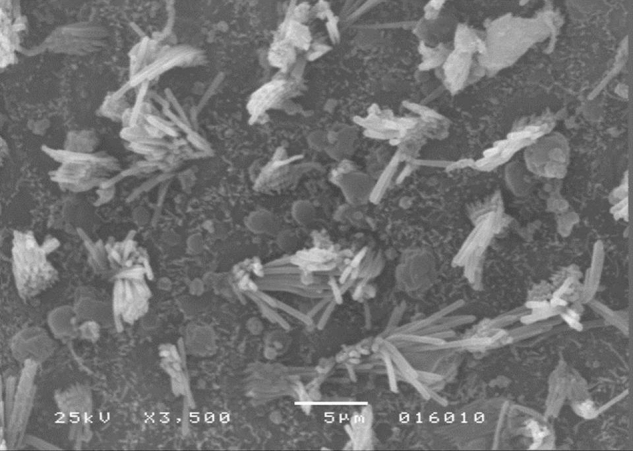
Saccule – Group I.

**Figure 3:**
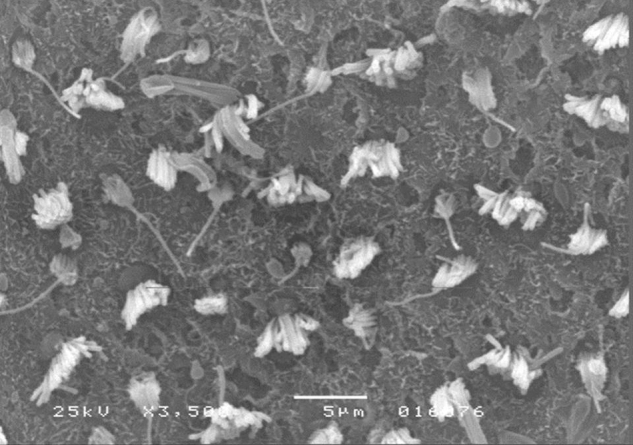
Utricle – Group II.

**Figure 4:**
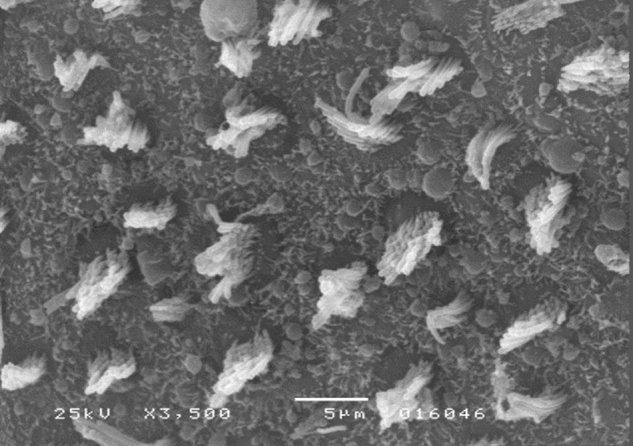
Saccule – Group II.

**Figure 5:**
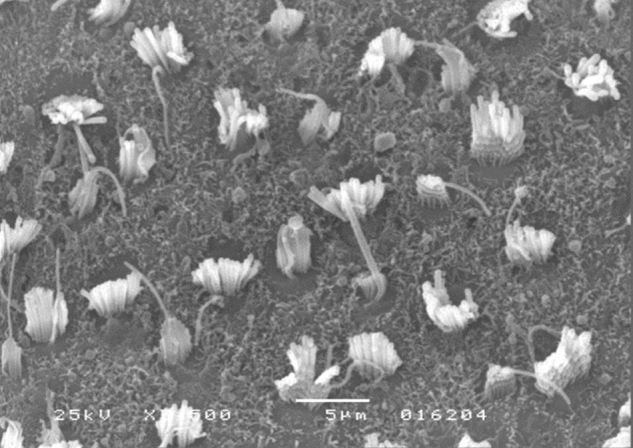
Utricle – Group III.

**Figure 6:**
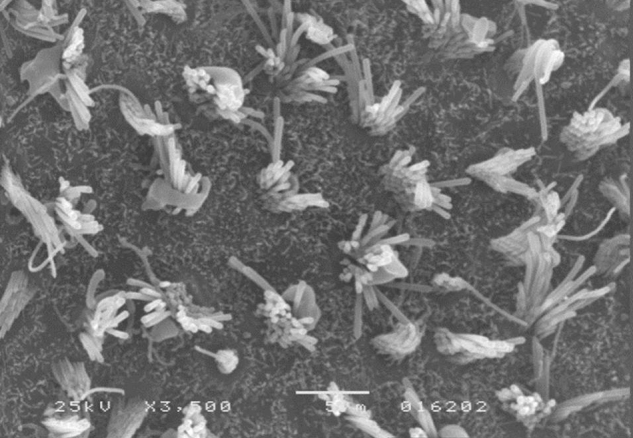
Saccule – Group III.

The results of comparative tests among the control group and the different doses of the organophosphate tested caused no histopathological damage to hair cells in saccules and utricles of guinea pigs involved in this study during the 10-day period, when analyzed by SEM (see Figure 1, Figure 2, Figure 3, Figure 4, Figure 5, Figure 6).

## Discussion

The mean for Group I was lower than that for the other groups, but it cannot be said that there was a significant difference among groups, because the means for Groups II and III are in line with mean values found by other authors, who utilized SEM in guinea pigs, one using distilled water for 30 days in their control groups, and another using saline fluid for 10 days.^20^, ^21^ The results of the present research showed no statistically significant difference between the numbers of ciliary tufts of groups of guinea pigs poisoned with different doses of an organophosphate pesticide. This is contrary to the findings of other investigators, who assessed the vestibular system of guinea pigs intoxicated by organophosphates for seven days, at doses of 0.3 and 3 mg/kg/day, and found ciliary changes in saccules and utricles.^22^ It is worth noting that their study used doses and an active agent different from those used in the present investigation.

The morphological damage in animals exposed to organophosphates has been widely researched. A study that acutely assessed the effects of an organophosphate in the nervous system observed structural change in the animals’ cerebellum, characterized by apoptosis of Purkinje cells and structural damage to the cytoskeleton of surviving animals.^23^ In a study that assessed chronic CNS effects in animals intoxicated by an organophosphate, hypertrophy of the molecular layer of the cerebral cortex was observed, which could lead to a loss or thinning of neural branches^24^, ^25^; however, this was not the objective of the present study, which evaluated ciliary tufts in sensory cells of saccule and utricle maculae.

A study on rural workers who came into contact with the organophosphate chlorpyrifos and later were submitted to tests for sensory-motor evaluation showed changes, especially in postural sway measures with closed eyes and on a soft surface. This result suggests a subclinical effect involving proprioceptive and vestibular systems.^10^

A functional assessment by vectoelectronystagmography of workers exposed to organophosphates revealed that the vast majority of the subjects showed irritant peripheral vestibular dysfunction-type changes.^9^ This finding emphasizes that organophosphate pesticides have neuro-ototoxic potential, but at present it is not possible to say what are the dose and duration of exposure considered as safe, in order to certify that these compounds will not cause harm to health.

Functional studies have demonstrated that pesticide poisoning causes dizziness and/or vertigo in workers exposed to these compounds,^8^, ^10^ but to date it has not been not possible to elucidate the specific morphological origin of this symptom, and even if there is a single cause or if this condition is multifactorial.

Further studies are needed to determine safe doses and exposure times for organophosphate pesticides. Therefore, it is noteworthy that at any dose and with any contact with these compounds, the use of suitable personal protective equipment is obligatory. Non-assessment of the semicircular canals was a limitation of the present study, and this should be considered in other studies; the subtleties of the anatomical structure were the factor that prevented their evaluation.

## Conclusion

Histopathological analysis of the vestibular system of guinea pigs exposed acutely to an organophosphate pesticide showed no difference in the amount of ciliary tufts in saccule and utricle maculae in the doses tested in this experiment. It is worth mentioning that the result for “saccule” variable was borderline, indicating that studies with a larger value of “n” may demonstrate significance.

## Conflict of interests

The authors declare no conflicts of interest.
